# Zebrafish and medaka T1R (taste receptor type 1) proteins mediate highly sensitive recognition of l‐proline

**DOI:** 10.1002/2211-5463.13558

**Published:** 2023-01-30

**Authors:** Ryusei Goda, Soichi Watanabe, Takumi Misaka

**Affiliations:** ^1^ Department of Applied Biological Chemistry, Graduate School of Agricultural and Life Sciences The University of Tokyo Japan; ^2^ Department of Aquatic Bioscience, Graduate School of Agricultural and Life Sciences The University of Tokyo Japan

**Keywords:** amino acid, l‐proline, medaka fish, taste receptor, zebrafish

## Abstract

In vertebrates, nutritional tastants, such as amino acids and sugars, are recognized by G‐protein‐coupled receptors of the taste receptor type 1 (T1R) family. Previous studies have shown that fish T1Rs are functionally distinct from mammalian T1Rs in certain regards. Here, we report the existence of oral receptors with high sensitivity to amino acids in zebrafish and medaka fish. We describe the construction of multiple cell lines stably expressing functional T1Rs (from medaka fish or zebrafish) with a chimeric G‐protein (G16gust44) using the Flp‐In system. Through functional assays with these cell lines, medaka fish and zebrafish were confirmed to possess particular T1Rs highly sensitive to l‐proline, possibly reflecting the physiological importance of l‐proline in teleosts, in line with previous studies.

## Graphical abstract

In this study, several cell lines expressing functional T1Rs (from medaka fish or zebrafish) with a chimeric G‐protein (G16gust44) were constructed using the Flp‐In system. Functional assays were conducted with the cell lines, and mfT1R2c/mfT1R3 and zfT1R2a/zfT1R3 were identified as highly sensitive taste receptors to l‐proline.

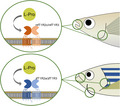

**Abbreviations**
∆RFU: delta relative fluorescent units
ANOVA: analysis of variance
DMEM: Dulbecco's modified Eagle's medium
EC_50_
: half maximal effective concentration
FBS: fetal bovine serum
GPCR: G‐protein‐coupled receptor
RFU: relative fluorescent units
SEM: standard error of the mean.
T1R: taste receptor type 1
T2R: taste receptor type 2

Taste is an important factor in determining the sensory characteristics of food products. In vertebrates, taste receptors belonging to the G‐protein‐coupled receptor (GPCR) family predominantly contribute to the detection of bitter, sweet, and umami tastants [1]. Bitter tastants, which include potentially toxic or harmful substances, are perceived through taste receptors type 2 (T2Rs) [2, 3, 4], and the activation of T2R‐expressing cells triggers aversive behavior [5, 6]. Alternatively, taste receptors type 1 (T1Rs) substantively mediate the recognition of natural substances with nutritional values, such as amino acids or sugars [4, 7, 8, 9]. Given the essential role of these nutrients in living organisms, the activation of T1R‐expressing cells evokes an appealing response [10].

The presence of taste receptor families has also been confirmed in fishes. In some fish species, such as zebrafish (*Danio rerio*), medaka fish (*Oryzias latipes*), threespine stickleback (*Gasterosteus aculeatus*), and rainbow trout (*Oncorhynchus mykiss*), T1Rs are expressed in multiple organs, such as the lips, palate, gill, pharynx, and barbels [4, 11, 12, 13, 14, 15]. Similar to mammalian T1Rs, fish T1R3 is mostly co‐expressed with either fish T1R1 or T1R2, although cells solely expressing T1R2 have also been reported [4, 11]. Moreover, previous studies on functional characterization using heterologous expression systems have demonstrated that fish T1Rs function as heterodimers of T1R1/T1R3 or T1R2/T1R3, similar to mammalian T1Rs, and that these receptors respond to diverse l‐amino acids [4, 16], although T1R2/T1R3 of herbivorous fish uniquely responds to sugars [17]. Recently, the crystal structure of the extracellular ligand‐binding domains of T1R2a, a T1R2 subtype, and the T1R3 heterodimer from medaka fish was reported, which provided a detailed structural insight into the broad amino acid recognition by the receptor [16].

Recent scientific evidence suggests that fish T1Rs are functionally distinct from mammalian T1Rs in certain regards. In mammals, T1R1/T1R3 respond to l‐amino acids and/or 5′‐ribonucleotides [8, 9, 18], whereas T1R2/T1R3 respond to sugars, d‐amino acids, as well as artificial sweeteners [7, 9, 19]. However, mammalian T1Rs generally exhibit low sensitivity to their natural ligands. For example, neither human T1R1/T1R3 response to l‐Glu nor that of human T1R2/T1R3 to sucrose saturated even at concentrations above 50 mm [20, 21]. By contrast, previously reported half maximal effective concentration (EC_50_) values of full‐length fish T1Rs to specific l‐amino acids ranged from tens to hundreds of micromolars [4, 16, 22]. Nevertheless, electrophysiological recordings from teleost fish have revealed threshold concentrations between 10^−7^ and 10^−9^ m against amino acids [23], suggesting the existence of specific oral recognition systems quite sensitive to amino acids.

This study clarifies the existence of oral receptors with high sensitivity to amino acids in zebrafish and medaka fish. As medaka fish and zebrafish are well‐known model organisms, a comprehensive analysis of their taste receptors will help us understand the fundamental mechanisms of the general taste recognition system. Several cell lines stably expressing functional fish T1Rs with a chimeric G‐protein (G16gust44) were constructed. Using these stable cell lines, the functional assays for the fish T1Rs were examined, and their responsiveness to low concentrations of l‐amino acids, especially to l‐Pro, was successfully characterized. The study results strongly indicate the existence of a T1R‐mediated, highly sensitive recognition system to l‐Pro in the two species.

## Materials and methods

### Sample solution and ligands

The assay buffer consisted of 10 mm 4‐(2‐hydroxyethyl)‐1‐piperazineethanesulfonic acid (HEPES), 130 mm NaCl, 10 mm glucose, 5 mm KCl, 2 mm CaCl_2_, and 1.2 mm MgCl_2_ (pH adjusted to 7.4 with NaOH). The ligands were diluted with the assay buffer at the desired concentrations.

Amino acids were obtained from commercial sources as follows. l‐cysteine, l‐aspartic acid sodium salt, l‐phenylalanine, glycine, l‐histidine monohydrochloride monohydrate, l‐lysine monohydrochloride, l‐proline (l‐Pro), l‐arginine (l‐Arg) hydrochloride, l‐serine, and l‐tyrosine were purchased from Nacalai Tesque (Kyoto, Japan); l‐alanine (l‐Ala), l‐isoleucine, l‐leucine, l‐asparagine monohydrate, l‐glutamine (l‐Gln), l‐threonine, l‐valine, and l‐tryptophane were purchased from Kanto Chemical (Tokyo, Japan); l‐glutamic acid monosodium salt was purchased from Sigma–Aldrich Japan (Tokyo, Japan); and l‐methionine was obtained from Tokyo Chemical Industry (Tokyo, Japan).

### Construction of the stable cell lines expressing functional fish T1Rs

All the cell lines used in this study were newly constructed in our laboratory as described below. For fish T1Rs, zebrafish and medaka fish T1Rs (mfT1R1, GenBank accession number AB200905; mfT1R2a, AB200906; mfT1R2b, AB200907; mfT1R2c, AB200908; mfT1R3, AB200909; zfT1R2a, AB200900; zfT1R2b, AB289806; and zfT1R3, AB200902) were used. Notably, some nucleotides were substituted in the cDNA clones used in this study. Guanine (G) at position 1180 was substituted with cytosine (C) (V394 to L394 for amino acids) for mfT1R2c, and C at position 741 was substituted with thymine (T) (Y247 to H247 for amino acids) for zfT1R3. As we used the T1R cDNA clones that were reverse‐transcribed from mRNA extracted from several individual fish [11], the substitution is likely a result of single‐nucleotide polymorphism.

**Fig. 1:**
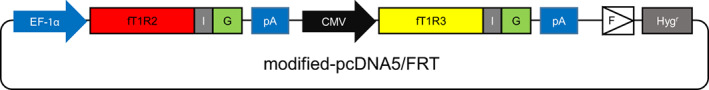
Expression construct of fish T1R2/fish T1R3 and G16gust44. Schematic illustration of the plasmid construction. F, FRT site; fT1R, the entire coding region of fish T1R; G, G16gust44; Hygr, hygromycin resistance gene; I, internal ribosome entry site (IRES) sequence; pA, polyadenylation signal.

Construction of the expression plasmids for generating stable cell lines was conducted as described previously [24], with a slight modification. The entire coding region of fish T1R1 (or T1R2), T1R3, and the chimeric G‐protein α‐subunit, G16gust44 [25], were subcloned into the pcDNA5/FRT vector (Thermo Fisher Scientific, Waltham, MA, USA) modified by a 6‐nucleotide mutation (Fig. 1). Flp‐In 293 cells (Thermo Fisher Scientific) were cultured at 37 °C in Dulbecco's modified Eagle's medium (DMEM) supplemented with 2 mm GlutaMax (Thermo Fisher Scientific) and 10% fetal bovine serum (FBS; Thermo Fisher Scientific). Cell lines were constructed using the Flp‐In pcDNA5/FRT complete system (Thermo Fisher Scientific) according to the manufacturer's protocol. Flp‐In 293 cells were transfected with the constructed expression plasmid and pOG44 (Thermo Fisher Scientific) for 48 h using Lipofectamine 2000 (Thermo Fisher Scientific). Then, the cells were selected by treatment with 100 μg·mL^−1^ hygromycin B for 2–3 weeks. Antibiotic‐resistant cells were collected, cultured, and used to measure the cellular response to l‐amino acids.

### Measurement of cellular responses by calcium imaging analysis

Measurements of the responses of cells stably expressing fish T1Rs by calcium imaging were conducted as described previously [24, 26] with a slight modification. Cells were seeded in 96‐well plates (Lumox Multiwell 96‐well, Starstedt AG, and Co., Nümbrecht, Germany) at approximately 50 000 cells per well. After 16–22 h, the medium was removed and replaced with assay buffer. Notably, the temperature of 37 °C, which is higher than that of the natural environment for fish, and/or desensitization caused by l‐amino acids in DMEM likely resulted in the degradation or the desensitization of the fish taste receptors. To improve the signal intensity by avoiding these problems, cells were incubated in the assay buffer at 27 °C for 3 h 15 min. After the incubation, cells were loaded with 5 μm fura‐2/AM (Thermo Fisher Scientific) in assay buffer for 30 min at 27 °C. The cells were washed with the assay buffer and incubated in 100 μL of assay buffer for 15 min at room temperature.

The cells were stimulated with l‐amino acids by adding 100 μL of 2× ligands at room temperature. The intensities of fura‐2 fluorescence emissions resulting from excitation at 340 and 380 nm were measured at 510 nm using a computer‐controlled filter exchanger (Lambda 10–3; Sutter Instruments, Novato, CA, USA), CoolSNAP HQ2 charge‐coupled device camera (Photometrics, Tucson, AZ, USA), and inverted fluorescence microscope (IX‐70; Olympus, Tokyo, Japan). The images were recorded at 4‐s intervals and analyzed using metafluor software (Molecular Devices, Sunnyvale, CA, USA). Data were presented in pseudocolor images based on the fluorescence intensity ratio at the two excitation wavelengths (F340/F380).

### Measurement of cellular responses using a cell‐based assay

Cellular responses were measured using a cell‐based assay as described previously [24, 26], with a slight modification. Cells were seeded in 96‐well plates (clear‐bottomed CellBIND surface plate, Corning, Glendale, AZ, USA) at approximately 70 000 cells per well. After 20 h, the medium was removed and replaced with assay buffer. After 3 h 15 min of incubation at 27 °C, cells were loaded with a calcium indicator dye from the FLIPR Calcium 4 Assay Kit (Molecular Devices) by dilution with the assay buffer. The cells were incubated for 45 min at 27 °C, and measurements were performed using FlexStation 3 (Molecular Devices) at 27 °C. Fluorescence changes (i.e., excitation at 485 nm and emission at 525 nm with a cutoff at 515 nm) were monitored at 2‐s intervals. A 100 μL aliquot of assay buffer supplemented with 2× ligands was added at 20 s, and scanning was continued for an additional 40 s.

The response of each well was determined as delta relative fluorescent units (∆RFU) and calculated as (maximum fluorescent value) – (minimum fluorescent value). The data are reported as the mean ± standard error of the mean (SEM) of the ∆RFU. Fitting curves for dose–response data and their correlation coefficient values were calculated by clampfit 10.4 (Molecular Devices) using Hill's equation. Statistical tests were conducted by ky plot version 6.0 (KyensLab Inc., Tokyo, Japan). A *P*‐value < 0.05 was considered statistically significant.

## Results

### The responsiveness of fish T1Rs to low concentrations of l‐amino acids

**Fig. 2:**
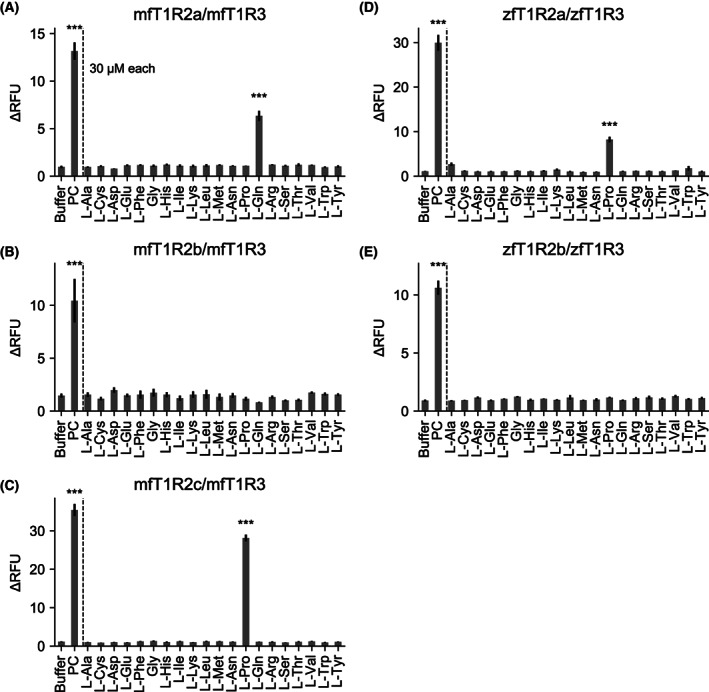
Response patterns of fish T1Rs to l‐amino acids and glycine. Quantification of the responses of cells stably expressing mfT1R2a/mfT1R3 (A), mfT1R2b/mfT1R3 (B), mfT1R2c/mfT1R3 (C), zfT1R2a/zfT1R3 (D), or zfT1R2b/zfT1R3 (E), with chimeric G‐protein (G16gust44), against 30 μm concentration of amino acids. “PC” refers to positive controls: 50 mm l‐Gln for mfT1R2a/mfT1R3, 50 mm l‐Pro for mfT1R2c/mfT1R3, and 50 mm l‐Ala for other receptors. Data were obtained by a cell‐based assay using flexstation 3. Each column represents the mean ± standard error of the mean (SEM) of at least three independent experiments. Significant differences in response to the buffer were analyzed using a one‐way analysis of variance (ANOVA) followed by Dunnett's test (***, *P* < 0.001). ∆RFU, Delta relative fluorescent units.

In this study, stable cell lines expressing fish T1Rs (mfT1R1/mfT1R3, mfT1R2a/mfT1R3, mfT1R2b/mfT1R3, mfT1R2c/mfT1R3, zfT1R2a/zfT1R3, or zfT1R2b/zfT1R3) with G16gust44 [25] were constructed as described in the Materials and methods. In stable cell lines generated using the Flp‐In system, a single expression vector is integrated at the specific location of the genome of Flp‐In 293 cells. As the expression level of the gene of interest is expected to be comparable within the stable cell lines with the same construct, more reliable examinations and comparisons are enabled. Additionally, the stable cell lines are convenient to prepare as a manual transfection procedure is not required once they are constructed. Given that the cell line expressing mfT1R1/mfT1R3 did not show obvious responses to the known ligand l‐Arg, [4] even at high concentrations (data not shown), it was excluded from subsequent experiments. However, other cell lines exhibited significant responses to known ligands (l‐Gln for mfT1R2a/mfT1R3, l‐Pro for mfT1R2c/mfT1R3, and l‐Ala for mfT1R2b/mfT1R3, zfT1R2a/zfT1R3, and zfT1R2b/zfT1R3; Fig. 2).

To explore the receptors highly sensitive to l‐amino acids, the constructed cell lines were stimulated with each of the 19 l‐amino acids or glycine at low concentrations (30 μm each, Fig. 2A–E). Consistent with previous publications [16, 22], mfT1R2a/mfT1R3‐expressing cells showed a significant response to 30 μm l‐Gln (Fig. 2A). Moreover, mfT1R2c/mfT1R3‐expressing cells (Fig. 2C) and zfT1R2a/zfT1R3‐expressing cells (Fig. 2D) responded significantly to 30 μm l‐Pro.

### Identification of the response thresholds of fish T1Rs to l‐amino acids

**Fig. 3:**
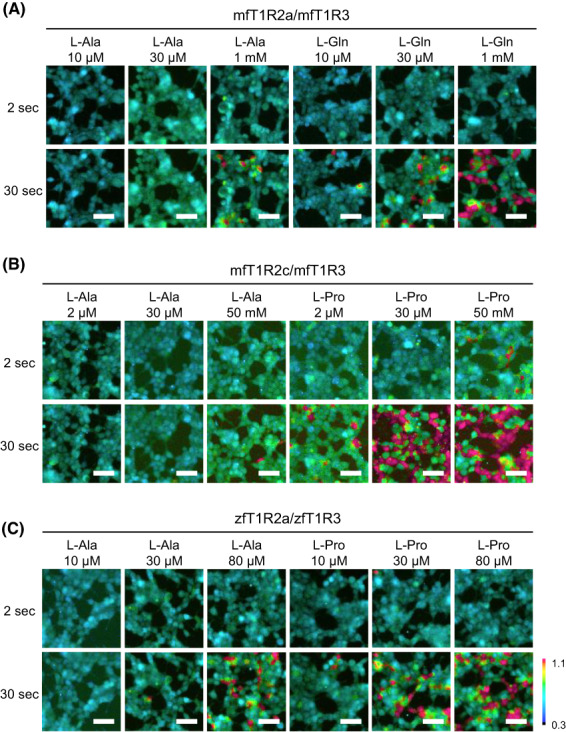
Response of fish taste receptor‐expressing cells to l‐amino acids. Representative ratiometric images of fura‐2 loaded cells stably expressing mfT1R2a/mfT1R3 (A), mfT1R2c/mfT1R3 (B), or zfT1R2a/zfT1R3 (C) with G16gust44. The upper and lower columns indicate images obtained approximately 2 and 30 s after stimulation, respectively. The color scale indicates the F340/F380 ratio as a pseudocolor. Scale bars: 50 μm.

Further analyses focused on mfT1R2a/mfT1R3, mfT1R2c/mfT1R3, or zfT1R2a/zfT1R3, all of which were characterized as the sensitive taste receptors to amino acids. To examine the threshold and specificity of the cellular responses to l‐Gln or l‐Pro by visualizing the cellular responses, Ca^2+^ imaging analyses were performed for stable cell lines expressing these receptors (Fig. 3). To compare the characteristics of the responses, l‐Ala was used as a control agonist.

The responses of mfT1R2a/mfT1R3 to 30 μm l‐Gln and those of mfT1R2c/mfT1R3 and zfT1R2a/zfT1R3 to 30 μm l‐Pro were observed (Fig. 3). The results were consistent with those in the cell‐based assay (Fig. 2) although the calcium indicator used in each method differed, indicating that the observed cellular responses to specific amino acids were not calcium indicator‐specific.

mfT1R2a/mfT1R3 responded to l‐Gln, even at 10 μm, and the number of responding cells dose‐dependently increased (Fig. 3A). Alternatively, mfT1R2c/mfT1R3 responded to low l‐Pro concentrations, with a ˂ 2 μm response threshold (Fig. 3B). Moreover, zfT1R2a/zfT1R3 responded to l‐Pro at concentrations of ≥ 10 μm (Fig. 3C). Although mfT1R2a/mfT1R3 and zfT1R2a/zfT1R3 responded to l‐Ala (Fig. 3A–C), their sensitivities to l‐Gln and l‐Pro were considerably high. Additionally, the responses of mfT1R2c/mfT1R3 to l‐Ala were obscure even at 50 mm. These results suggest l‐Pro as a possible potent agonist of fish T1Rs, such as mfT1R2c/mfT1R3 and zfT1R2a/zfT1R3.

### Concentration‐dependent response of fish T1Rs to specified l‐amino acids

**Fig. 4:**
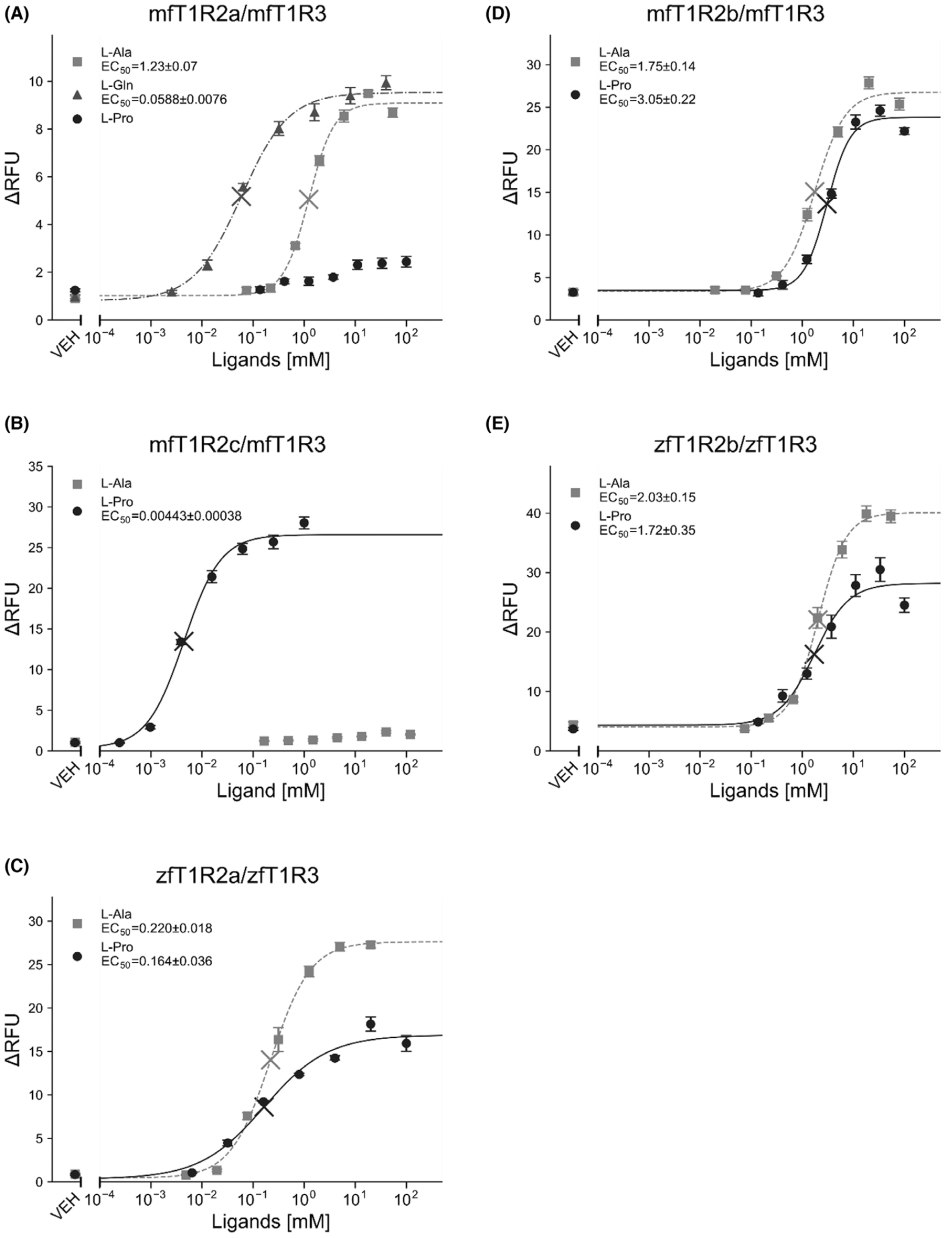
Dose‐dependent responses to l‐amino acids of the fish taste receptor‐expressing cells. Dose–response curves for l‐amino acids were determined for stable cell lines expressing mfT1R2a/mfT1R3 (A), mfT1R2c/mfT1R3 (B), zfT1R2a/zfT1R3 (C), mfT1R2b/mfT1R3 (D), or zfT1R2b/zfT1R3 (E) with G16gust44. Data are presented as the mean, and error bars indicate the SEM from four independent experiments. Cross marks in the graph represent EC_50_ values. ∆RFU, Delta relative fluorescent units; VEH, responses against buffer.

To quantify the sensitivity of the receptors, dose–response curves for the specified l‐amino acids were examined using a cell‐based assay (Fig. 4). This experiment also revealed that l‐Pro could be a potent agonist of fish T1Rs, similar to the results of Ca^2+^ imaging analyses (Fig. 3).

mfT1R2c/mfT1R3 responded to l‐Pro at a concentration of ˂ 1 μm and exhibited a dose‐dependent increase in response to l‐Pro with an EC_50_ value of 4.43 ± 0.38 μm (Fig. 4B). This concentration was quite low compared to that of mfT1R2a/mfT1R3 response to l‐Gln, whose EC_50_ value was 58.8 ± 7.6 μm (Fig. 4A). The responses of mfT1R2c/mfT1R3 to l‐Ala at > 4.44 mm were statistically significant against the buffer [one‐way analysis of variance (ANOVA) followed by Dunnett's test; *P* < 0.05 for 4.44 mm, *P* < 0.01 for 13.3 mm, *P* < 0.001 for 40 and 120 mm], indicating that mfT1R2c/mfT1R3 responded to high concentrations of l‐Ala, consistent with the result of a previous study [4]. Since the maximum signal intensity against l‐Ala (at 40 mm) was only twice that against the buffer, we doubt the reliability of fitting values, and the dose–response curve for l‐Ala is not shown (Fig. 4B).

EC_50_ values of mfT1R2a/mfT1R3 response to l‐Ala (1.23 ± 0.07 mm) and l‐Gln (58.8 ± 7.6 μm) were similar to those reported in previous publications [4, 16, 22], indicating the reliability of the constructed cell lines stably expressing fish T1R2/T1R3 in this study. Additionally, the responses of zfT1R2a/zfT1R3 were also dose‐dependent, with EC_50_ values of 220 ± 18 μm (to l‐Ala), consistent with our previous report [4], and 164 ± 36 μm (to l‐Pro). The responses of mfT1R2a/mfT1R3 to L‐Pro only at 11.1 mm or higher were statistically significant against the buffer in a cell‐based assay [one‐way analysis of variance (ANOVA) followed by Dunnett's test; *P* < 0.001], indicating that the responses against l‐Pro at least at ~ mm were definitely receptor specific (Fig. 4A). Additionally, the maximum signal intensity of mfT1R2a/mfT1R3 against l‐Pro (at 100 mm) was considerably smaller than that of other fish taste receptors examined in this study, suggesting that the strong responses to l‐Pro at > 10 mm were also receptor specific. Fitting values of mfT1R2a/mfT1R3 for l‐Pro were not shown for the same reason as mfT1R2c/mfT1R3 for L‐Ala.

Although mfT1R2b/mfT1R3 and zfT1R2b/zfT1R3 did not respond to l‐Pro at 30 μm in this study (Fig. 2B,E), their responses to l‐Pro and l‐Ala at 50 mm were reported in a previous study [4]. Therefore, their concentration‐dependent responses to l‐Ala and l‐Pro were also examined. In contrast to mfT1R2c/mfT1R3 and zfT1R2a/zfT1R3, EC_50_ values of mfT1R2b/mfT1R3 and zfT1R2b/zfT1R3 to l‐Pro were 3.05 ± 0.22 and 1.72 ± 0.35 mm, respectively (Fig. 4D,E). This result emphasizes the high sensitivity of mfT1R2c/mfT1R3 and zfT1R2a/zfT1R3 to L‐Pro. Moreover, EC_50_ values of mfT1R2b/mfT1R3 and zfT1R2b/zfT1R3 to L‐Ala were 1.75 ± 0.14 and 2.03 ± 0.15 mm, respectively, which are comparable with the results of a previous study [4].

## Discussion

Receptors with high sensitivity to l‐Pro, such as mfT1R2c/mfT1R3 and zfT1R2a/zfT1R3, were successfully characterized in this study (Figs 2, 3, 4). Accordingly, facial nerve recording analyses of zebrafish indicated a strong response to l‐Pro [4]. The preference of medaka fish for a mixture of amino acids, including l‐Pro, has also been confirmed through behavioral experiments [27]. Additionally, several pieces of evidence have been proposed for other fish species, in which l‐Pro elicited a taste response at the lowest concentration among all l‐amino acids [28, 29, 30], suggesting that the highly sensitive features of T1Rs to l‐Pro are conserved among teleost fishes.

The characteristics of taste perception in animals often reflect their nutrient requirements. Proline is a conditionally indispensable amino acid in some fish species, suggesting the importance of dietary l‐Pro in maintaining appropriate metabolic conditions in teleosts [31, 32, 33, 34]. Therefore, selective intake of feed rich in l‐Pro may be advantageous for meeting the nutrient requirements of fishes. Medaka fish and zebrafish are freshwater and omnivorous species that consume a wide range of feeds, such as small arthropods, zooplankton, zoobenthos, and phytoplankton [35, 36]. The most abundant medaka fish prey in the natural environment is zooplankton [37]; however, the free form to total l‐Pro ratio is low in freshwater zooplankton [38]. The high sensitivity of T1Rs to l‐Pro can contribute to the effective selection of Pro‐rich feeds for medaka fish and zebrafish in their natural habitats. Moreover, the content of free l‐Pro considerably decreases in fasted *Artemia salina nauplii*, living in inland saltwater lakes, and marine copepod *Temora longicornis*, whereas those of essential amino acids are stable [38, 39], suggesting that l‐Pro is a good indicator of the nutritional value of fish prey. Similar trends have also been reported in the plasma of freshwater prawn *Macrobrachium rosenbergii* [40], though their larvae inhabit brackish water. The high sensitivity of T1Rs to l‐Pro could contribute to the effective selection of nutritional feeds for medaka fish and zebrafish. However, there are few works describing the relationships between free amino acids' compositional change and the nutritional value of freshwater zooplankton. Further research into the roles of l‐Pro on freshwater zooplankton is needed to clarify the physiological importance of the characteristic of fish taste responses.

Regarding ligand sensitivity, the EC_50_ value of mfT1R2c/mfT1R3 response to l‐Pro was 4.43 μm (Fig. 4B), which is similar to that of mGluRs, representative members of the class C GPCR family, to l‐Glu, except for mGluR7 [41], and the detection threshold concentration of l‐Pro was ˂ 2 μm (Fig. 3B). However, this value is still higher than the threshold concentration determined by electrophysiological recordings from teleost fish (10^−7^ ~ 10^−9^ m) [23]. Consistent with this observation, the l‐Ala threshold in the nerve recordings from zebrafish in our previous report (approximately 10 nm) was lower than that obtained by the heterologous expression system in both this study and the previous study [4]. This discrepancy may be because of the use of chimeric G‐protein (G16gust44), which does not exist in actual fish taste receptor cells. In fact, although zfT1R1/zfT1R3 does not respond to any l‐amino acids when co‐expressed with chimeric G‐protein [4], *t1r1*‐KO zebrafish showed significantly reduced preference for L‐Ala, suggesting a role for zfT1R1 in feeding behavior in the natural environment [42]. In zebrafish, the absence of an ortholog of mammalian gustducin and expression of other G‐proteins have also been reported [43, 44].

In a previous report, mfT1R2a/mfT1R3 responded to a broad spectrum of amino acids, whereas mfT1R2c/mfT1R3 response to l‐amino acids was limited just to l‐Ala or l‐Pro [4]. In this study, mfT1R2c/mfT1R3 exhibited much weaker responses to l‐Ala than to l‐Pro, even at high concentrations (Figs 3B and 4B), further indicating its high specificity to l‐Pro, in contrast to mfT1R2a/mfT1R3. Previous crystallographic analyses revealed that the ligand‐binding pocket of mfT1R2a is rich in aromatic residues and has a large space [16]. Different ligands are surrounded by water molecules in this spacious pocket, and they bind indirectly to the receptor through water‐mediated hydrogen bonds, possibly explaining the diverse ligand perceptions of mfT1R2a/mfT1R3 [16]. Regarding the ligand recognition‐related residues, discrepancies were observed between mfT1R2a and mfT1R2c. mfT1R2a S165, which directly binds to l‐amino acids and whose mutation to Ala results in a weakened or no response to l‐amino acids [16], is actually replaced by Ala in mfT1R2c. Additionally, mfT1R2a D288, which indirectly binds to the ligands, is also replaced by Ala in mfT1R2c. The high specificity and sensitivity of mfT1R2c/mfT1R3 may be attributed to these residues. However, mfT1R2a D288 is also replaced by Ala in mfT1R2b, which responded to a wide range of l‐amino acids when co‐expressed with mfT1R3 [4]. Given that key residues for the broad response of mouse T1R1/T1R3 exist outside the ligand‐binding site [21], the difference in ligand specificity among fish T1Rs may also depend on residues at nonorthosteric sites.

Although zfT1R2a/zfT1R3 exhibited higher sensitivity to l‐Pro than l‐Ala, l‐Ala was more potent in terms of efficacy (Fig. 4C). Notably, the features of zfT1R2b/zfT1R3 responses to these l‐amino acids were the same (Fig. 4E), although its EC_50_ values to both ligands were approximately 10 times higher than those of zfT1R2a/zfT1R3. l‐Pro is unique because it is the only cyclic amino acid among the 20 natural α‐amino acids. The α‐amino group of several l‐amino acids reportedly contributes to the recognition by mfT1R2a/mfT1R3 by directly binding to mfT1R2a S165 side‐chain hydroxyl and main‐chain carbonyl groups [16]. Considering the specific structure, a strategy distinct from the case in other l‐amino acids might be required to recognize l‐Pro, which may explain the difference in efficacy. However, the efficacies of l‐Ala and l‐Pro on mfT1R2b/mfT1R3 were almost comparable (Fig. 4D). The recognition manner of l‐Pro by T1Rs and its relationship between ligand efficacies are important points for future studies.

In conclusion, cell lines stably expressing functional fish T1Rs with G16gust44 were constructed, and the study results demonstrated that a certain type of T1R in zebrafish and medaka fish could detect l‐Pro at low concentrations.

## Conflict of interest

The authors declare no conflict of interest.

## Author contributions

TM and SW conceived and supervised the study; RG designed and performed experiments, and analyzed data; RG, SW, and TM wrote the manuscript.

## Data Availability

The data that support the findings of this study are available from the corresponding author [amisaka@mail.ecc.u-tokyo.ac.jp] upon reasonable request.
